# Biocontrol of *Staphylococcus aureus* in dairy products using lytic bacteriophage UHP46: efficacy in milk, cheese, and yogurt

**DOI:** 10.1007/s42770-026-02081-w

**Published:** 2026-09-24

**Authors:** Sara Najeeb, Imran Khan, Javed Muhammad, Muhammad Jahangir, Anza Abbas, Aman Ullah, Amjad Khan, Sumaira Miskeen

**Affiliations:** 1 Department of Food Science and Technology, The University of Haripur, Khyber Pakhtunkhwa, Pakistan; 2 Research Institute of Nutrition and Food Safety, Universitat de Barcelona, Santa Coloma de Gramenet, 08921 Spain; 3 Department of Microbiology, The University of Haripur, Khyber Pakhtunkhwa, Pakistan; 4 Department of Microbiology, Afghan International Islamic University, Kabul, Afghanistan; 5 Department of Veterinary Sciences, University of Kentucky, Lexington, KY USA; 6 Department of Food Science and Nutrition, Women University Swabi, Khyber Pakhtunkhwa, Pakistan

**Keywords:** Lytic bacteriophage, *Staphylococcus aureus*, Antimicrobial resistance, Phage biocontrol, Dairy food safety, Staphylococcal enterotoxin

## Abstract

*Staphylococcus aureus* is a major public health concern in the dairy industry. It is a well-recognized pathogen responsible for bovine mastitis and raw milk contamination; certain strains produce heat-stable enterotoxin capable of causing staphylococcal food poisoning in humans. The emergence of multiple drug-resistant strains of *S. aureus* has rendered ineffective the use of antibiotics for control purposes. This study determined the antibacterial activity of lytic bacteriophage UHP46 against a single, previously characterized multidrug-resistant mastitis isolate, *S. aureus* S46, in three dairy matrices — milk, cheese and yogurt — under storage conditions representing refrigerated storage (4 °C), ambient conditions (25 °C) and a 37 °C mechanistic reference condition. UHP46 significantly reduced *S. aureus* S46 counts in milk, achieving reductions of 3.2 and 3.7 log₁₀ CFU/mL after 4 and 24 h at 4 °C, 2.1 and 3.1 log₁₀ CFU/mL after 4 and 24 h at 25 °C, and 1.8 log₁₀ CFU/mL after 3 h at 37 °C. The reduction at 37 °C was accompanied by an increase in phage titer from 7.2 to 8.8 log₁₀ PFU/mL. Application of UHP46 to cheese resulted in an immediate reduction of 0.6 log₁₀ CFU/g after 5 min of treatment at room temperature. The maximum reduction observed was 2.0 log₁₀ CFU/g at 24 h, with a sustained reduction of 1.9 log₁₀ CFU/g maintained through 96 h of refrigerated storage at 4 °C. In yogurt, net reductions on day 6 were 2.8, 2.7, and 1.6 log₁₀ CFU/g at 37 °C, 25 °C, and 4 °C, respectively. The findings indicate that UHP46 exhibits significant antibacterial activity against *S. aureus* S46 within the tested dairy matrices and under the evaluated storage conditions. However, as all challenge experiments utilized a single host strain, its efficacy against a broader panel of *S. aureus*, including methicillin-resistant *S. aureus* (MRSA), remains to be determined.

## Introduction

*S. aureus* poses a significant challenge in dairy production systems, responsible for bovine mastitis, low milk production, drug resistance, and dissemination of antimicrobial resistance within the food production chain [36, 42]. The main challenge with controlling the disease-causing agent within the milk production process is its ability to generate heat-stable Staphylococcal enterotoxins (SEs), which can still be present even when the bacterial load has been reduced to acceptable levels [24]. This poses a persistent threat not only to the health of the host animal but also to the consumers of milk-derived products [8]. Dairy production systems occupy an important role in global food security, supplying substantial nutritional benefits worldwide while simultaneously making milk and dairy products susceptible to foodborne bacterial pathogens at different stages of the dairy supply chain (OECD & FAO, [31]). *S. aureus* is one of the most prevalent and clinically significant bacteria that cause bovine mastitis [42]. The pathogenicity of *S. aureus* inside the udder is mediated through an advanced set of virulence factors that include microbial surface components recognizing adhesive matrix molecules (MSCRAMMs), secretory enzymes, biofilm-related proteins, and toxins [5, 25]. These virulence factors work together to degrade udder tissue, decrease milk production, and induce systemic inflammation in affected animals [5]. The public health relevance of *S. aureus* is mostly related to SE, which are globular proteins that are small, extremely stable, and resistant to heat, proteolysis, and wide pH ranges. These proteins have been linked to outbreaks of staphylococcal foodborne illnesses all over the world [3]. These foods cause food poisoning along with immediate symptoms such as nausea, vomiting, and abdominal cramps [6]. Heat treatment destroys vegetative bacteria, but does not neutralize enterotoxins that have already been produced unless it is done properly; therefore, prevention is better than anything else [30]. Another issue that has made controlling the infection caused by *S. aureus* in the dairy industry more challenging is the emergence of antimicrobial resistance throughout the world [27]. Additionally, the identification of MRSA in milk samples has highlighted the importance of this problem within the One Health paradigm because the resistant genes and bacteria may spread throughout the animal-human environment interface and cause issues through the food chain [7].

Nevertheless, the approaches traditionally employed to control *S. aureus* in dairies have certain limitations, which include antibiotic usage, chemical preservation, and pasteurization/thermal treatments. Thermal treatments such as pasteurization enhance safety from microorganisms but may cause changes in protein composition, affecting flavor, smell, and even the texture of dairy products [33]. Chemical preservation is becoming more controversial in recent times due to clean-label preferences, and new technologies require special equipment and training, which may not be possible for all dairy operators [38]. Thermal treatment cannot completely remove all possible risks due to post-processing contamination and biofilm-mediated persistence [20, 37]. These limitations continue to drive interest in bio-preservation techniques targeting specific, clean-label processes such as the use of lactic acid bacteria and their products as well as phages, as a supplementary measure to existing methods [10]. The ability of bacteriophages to target and destroy their hosts without disrupting other components in their immediate environments makes them highly promising candidates as biocontrol agents in food systems [18, 34]. This process is made possible by the phage’s capacity to bind with target receptors, insert themselves into the host cell, commandeer host cellular machinery for replication, ultimately lysing the cell and releasing progeny phage particles [13, 39]. In fermented food systems, where maintaining lactic acid bacteria is essential, this specialty is particularly essential [18, 34]. Bacteriophages have undergone evaluation through the U.S. Food and Drug Administration’s Generally Recognized as Safe (GRAS) notification process for specific food applications, including formulations containing phages targeting Shiga-toxin-producing *Escherichia coli* (U.S. Food and Drug Administration) [43].

The use of phage for biocontrol of *S. aureus* has been studied in a few dairy-related matrices, but mostly in isolation. In pasteurized milk, lytic phages like phiIPLA35 and phiIPLA88 and KMSP1 have been found to be effective in reducing *S. aureus* counts, and their efficiency appears to be influenced by the ratio of phages to hosts and the incubation temperature [14, 21]. In cheese, phage cocktails have demonstrated reductions of over 4 log₁₀ CFU/g in the experimentally contaminated hard cheese although a limitation of phage cocktails is bacterial regrowth in the cheese over extended storage, which is believed to be related to limited diffusion of phages within the solid matrix and decreased access to the bacteria hidden in the cheese [4, 9]. Although fermented dairy products such as yogurt are more difficult to study because of the low pH, which can affect the stability of phages and/or their infectivity, similar studies are also comparatively few in number. Most of these previous studies have focused on one food matrix in a small range of storage temperatures, with little study of how the interaction of food matrix and temperature affects phage performance [40].

Multiple biological and physicochemical factors influence phage performance in food systems, each directly affecting the interpretation of matrix- and temperature-dependent outcomes. First, the kinetics of lytic infection, including adsorption rate, latent period, and burst size, are determined by the physiological state of the host and, consequently, by temperature. Replication parameters typically improve as environmental conditions approach the optimal growth temperature of the host bacterium [17, 19]. Temperature also governs host multiplication, so the net antibacterial outcome observed in a food system is determined by the balance between phage-mediated killing and bacterial growth rather than by infection kinetics alone. Refrigeration slows phage replication but also restricts host growth, and phage efficacy has been reported to increase under conditions that limit bacterial multiplication [12]. Second, exposure to a lytic phage imposes strong selection for phage-insensitive derivatives of the host population. In *S. aureus*, insensitivity most commonly arises through modification or loss of the wall teichoic acid structures that serve as adsorption receptors [44]. Constitutive defense systems such as restriction–modification and abortive infection can further restrict propagation [22]. The rate at which insensitive subpopulations become detectable is itself temperature-and physiology-dependent [11]. Therefore, the occurrence of bacterial regrowth in a treated food does not, by itself, prove acquired bacterial resistance, as it may be due to the presence of surviving bacteria in spatial or physiological refuges. Third, phage particles exhibit limited diffusion through a structured protein–fat network, which restricts contact with bacteria embedded within the matrix. As a result, inactivation of the bacterial population remains incomplete, permitting surviving bacteria to resume growth during extended storage [4, 9]. Finally, fermented dairy products have a characteristically low pH (normally 4.0-4.5), which can compromise phage stability and infectivity [40]; UHP46 had been previously found to be infective at pH 4-10, and this was the basis for testing it in yogurt in this study [26].

This study evaluates the lytic phage UHP46 in three dairy matrices—milk, cheese, and yogurt—under three temperature conditions: refrigerated (4 °C), ambient (25 °C), and mechanistic reference (37 °C). It provides a comparative assessment of UHP46 performance across these matrices and storage conditions.

## Materials and methods

### Isolation and identification of *S. aureus*

*S. aureus* S46 was isolated and identified as previously described [26]. In accordance with National Mastitis Council [29] guideline, milk samples were aseptically obtained from dairy farms in Khyber Pakhtunkhwa, Pakistan. Samples testing positive by the California Mastitis Test (CMT) underwent microbiological analysis. *S. aureus* was enriched in Brain Heart Infusion broth at 37 °C for 24 h and then plated on Mannitol salt agar (MSA) [15]. Identification was confirmed through Gram staining and biochemical assays, such as the Voges-Proskauer (VP), coagulase, catalase, urease, indole, and methyl red tests [41]. The phenol-chloroform isoamyl alcohol method was used to extract genomic DNA, and molecular identification was performed via 16S rDNA sequence analysis [35]. The Kirby-Bauer disk diffusion assay was used to determine antibiotic susceptibility in compliance with CLSI 2019 guidelines [16].

### Isolation and characterization of bacteriophage

Bacteriophage UHP46 was isolated and characterized as previously described [26]. Morphological analysis was conducted using transmission electron microscopy (TEM). The pH and thermal stability of the phage were assessed by exposing samples to pH values ranging from 2 to 12 and temperatures from 20 to 85 °C. The host range was evaluated against 29 *S. aureus* strains isolated from bovine mastitis milk using the double agar overlay method. UHP46 lysed 4 of the 29 tested *S. aureus* strains (13.8%), indicating a narrow host range. The phage-only stability in heat-treated milk was assessed at 4 °C, 25 °C, and 37 °C, with titers determined by double-layer agar assay at 4 h and 24 h. A high-titer stock (1 × 10^9^ PFU/mL) was freshly prepared and verified by plaque assay immediately before experimental use.

### Determination of the phage lytic activity in milk

Phage UHP46 activity was assessed in raw milk sterilized by direct exposure to high temperature (110 °C for 20 min) followed by UV-C irradiation at 253 nm for 15 min to eliminate background microbiota. Prior to experimentation, sterility was confirmed by plating milk samples on MSA; the absence of bacterial growth indicated no contaminating flora. Sterile tubes containing 5 mL of milk were inoculated with *S. aureus* at a final concentration of 1 × 10⁵ CFU/mL. This inoculum level was chosen because it closely reflects *S. aureus* concentrations typically found in raw milk from cows with subclinical or clinical mastitis and is commonly used in phage challenge assays, enabling reliable enumeration and clear assessment of phage-mediated reductions. Inoculated samples were allocated to two groups: an untreated control and a phage-treated group (UHP46 at 1 × 10⁷ PFU/mL), corresponding to a multiplicity of infection (MOI) of approximately 100. Tubes were incubated at 37 °C with shaking for 3 h. At 0, 90, and 180 min, 100 µL aliquots were collected, serially diluted, and plated on MSA to quantify bacterial counts (CFU). Phage titers (PFU/mL) were measured before and after incubation using the double agar overlay method on Nutrient Agar (NA). Prior to phage titration, milk samples were filtered through 0.22 μm syringe filters to remove bacterial cells and debris, ensuring that plaque counts reflected only free phage particles. Incubation at 37 °C was used as a mechanistic reference condition, corresponding to the optimal growth temperature of *S. aureus*. This approach facilitated evaluation of the intrinsic lytic activity and replication capacity of UHP46 under conditions of maximal adsorption and replication kinetics. However, 37 °C does not represent typical milk storage or handling temperatures. Consequently, the phage was also introduced into 5 mL of heat-treated milk at a final concentration of 1 × 10⁷ PFU/mL to evaluate bacterial survival at 25 °C and 4 °C for 4 and 24 h [1]. The 25 °C and 4 °C conditions were selected to simulate ambient temperature-abuse and refrigerated storage scenarios, respectively.

### Effect of UHP46 on experimentally contaminated cheese

A solid pasteurized cheese sample was obtained from a local market in Haripur, Khyber Pakhtunkhwa, Pakistan. A 50 g portion was loosely covered and inoculated on all surfaces with approximately 1 × 10⁵ CFU/g of *S. aureus* S46 by evenly pipetting and spreading the bacterial suspension across the entire cheese surface. The sample was maintained at room temperature for 60 min to facilitate bacterial adhesion. The cheese was then divided into two 25 g portions and treated at a rate of 1 mL per 100 g of cheese (0.25 mL per portion). The control group received sterile PBS, while the treated group was administered UHP46 suspension at a titer of 1 × 10⁹ PFU/mL, delivering 2.5 × 10⁸ PFU per portion. Treatment solutions were evenly pipetted and spread over the entire surface of each cheese portion to ensure uniform distribution. This resulted in a final dose of 1 × 10⁷ PFU/g of cheese, corresponding to a MOI of 100 relative to the *S. aureus* S46 inoculum of 1 × 10⁵ CFU/g. Samples were covered and incubated at room temperature for 5 min. A 5 g sample from each group was transferred to separate sterilized stomacher bags containing 45 mL of sterile peptone water and processed for 30 s. The remaining cheese was stored at 4 °C and sampled in triplicate at 24 and 96 h post-treatment. The number of viable *S. aureus* cells was determined by preparing ten-fold serial dilutions in buffered peptone water (pH 7.0), plating onto MSA, and incubating at 37 °C for 24 to 48 h. Viable counts were reported as log₁₀ colony-forming units per gram (log₁₀ CFU/g) [32].

### Evaluation of bacteriophage activity in yogurt

The antibacterial activity of UHP46 against *S. aureus* S46 was assessed in yogurt under different storage temperatures. Plain whole-milk yogurt, commercially produced and sourced from a local market in Haripur, Khyber Pakhtunkhwa, Pakistan, was stored at 4 °C and utilized within 24 h of opening. An overnight culture of *S. aureus* S46 was grown in Nutrient broth to approximately 2 × 10⁸ CFU/mL and serially diluted to achieve a target inoculum of approximately 2 × 10³ CFU/g for yogurt contamination. The inoculated yogurt was incubated at room temperature for 60 min to permit *S. aureus* S46 cells to equilibrate within the yogurt matrix prior to phage treatment. For the treatment group, 10 mL of UHP46 suspension (2 × 10⁶ PFU/mL) was added to 100 mL of yogurt, resulting in 2 × 10⁷ PFU and a final phage concentration of approximately 1.8 × 10⁵ PFU/mL. Relative to the *S. aureus* S46 inoculum (approximately 2 × 10⁵ CFU total), this yielded a MOI of 100. The untreated control received an equal volume of sterile distilled water. All samples were stored at 37 °C, 25 °C, and 4 °C for six days, with bacterial counts determined on days 2, 4, and 6. Three replicate subsamples (25 g each) were aseptically transferred into stomacher bags containing 225 mL of sterile peptone water (1:10, w/v) and homogenized for 30 s. Serial ten-fold dilutions were prepared, plated in duplicate on MSA, and incubated at 37 °C for 24 to 48 h. Bacterial populations were reported as log₁₀ CFU/g [28].

### Statistical analysis

Data from the milk and cheese experiments were analyzed using two-way analysis of variance (ANOVA) to assess the effects of treatment, storage conditions, and their interaction. In the yogurt experiment, one-way ANOVA was conducted to compare bacterial reductions across different storage temperatures. Tukey’s multiple-comparison test was applied for post hoc analyses where appropriate. Statistical significance was defined as *p* < 0.05.

## Results

### Antibacterial activity of phage UHP46 in milk

**Fig. 1:**
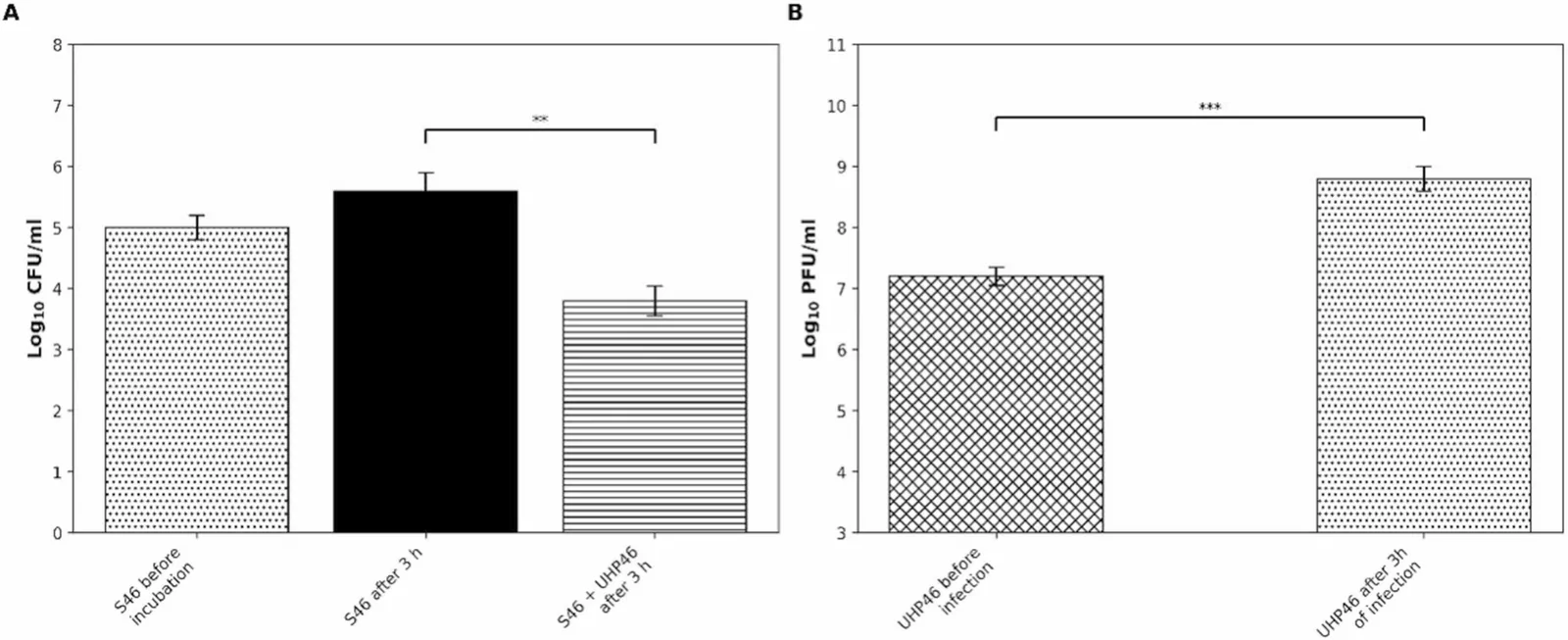
Antibacterial activity of bacteriophage UHP46 in milk at 37 °C. (**A**) Reduction in *S. aureus* S46 counts (log₁₀ CFU/mL) after 3 h of incubation in control and phage-treated samples. (**B**) Phage titers (log₁₀ PFU/mL) measured before infection and after 3 h, showing UHP46 replication in milk

**Fig. 2:**
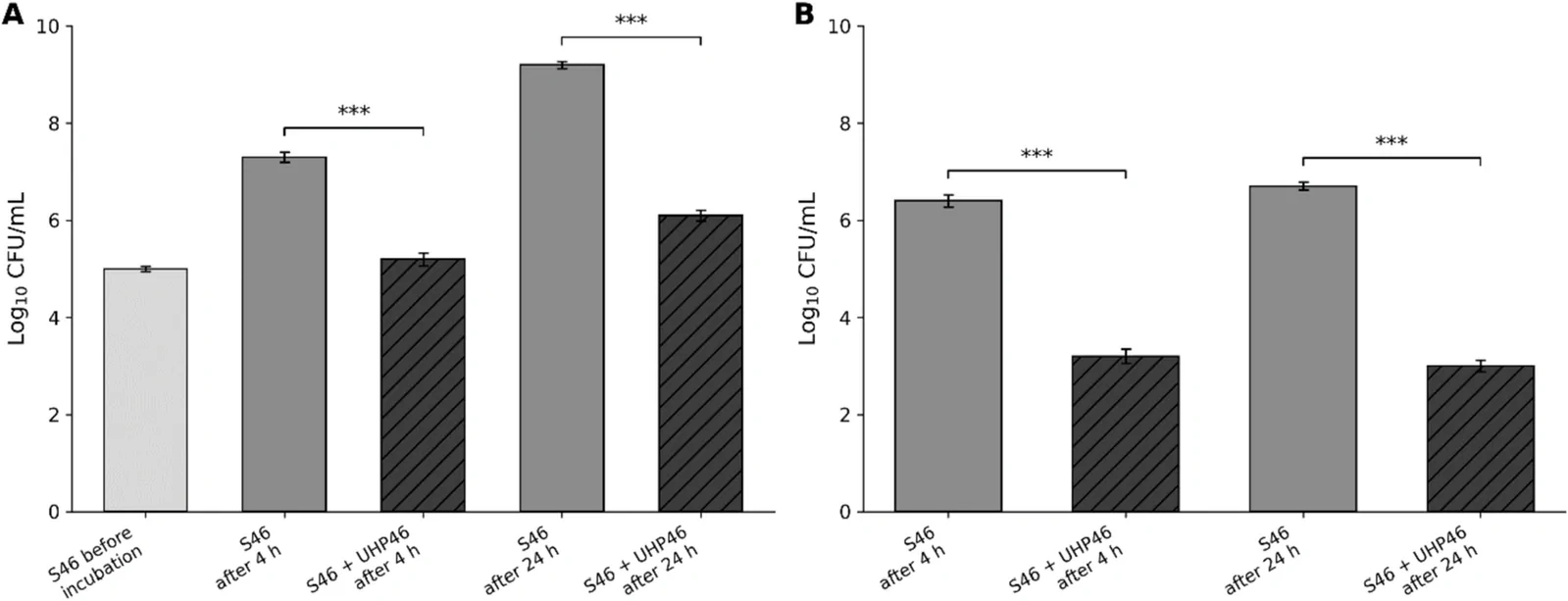
Inhibitory effect of bacteriophage UHP46 on *S. aureus* S46 in milk at different temperatures. (**A**) Bacterial counts (log₁₀ CFU/mL) at 25 °C measured prior to incubation, and after 4 and 24 h of incubation with or without UHP46. (**B**) Bacterial counts at 4 °C (refrigeration temperature) measured prior to incubation, and after 4 and 24 h of incubation with or without UHP46

The influence of phage UHP46 on bacterial growth in milk was studied under ideal conditions by performing an infection test at 37 °C (Fig. 1A). In the absence of phage treatment, bacterial counts increased from 5.0 to 5.6 log₁₀ CFU/mL after 3 h of incubation. In contrast, phage-treated samples exhibited a reduction in bacterial counts to 3.8 log₁₀ CFU/mL, corresponding to a 1.8 log₁₀ CFU/mL decrease compared to the untreated control (*p* < 0.05). Phage propagation was evaluated under the same conditions (Fig. 1B). The phage titer increased significantly from 7.2 to 8.8 log₁₀ PFU/mL after 3 h of incubation at 37 °C (*p* < 0.05), demonstrating active replication of phage UHP46 in the presence of its bacterial host. To evaluate the efficacy of phage UHP46 at ambient temperature (25 °C), bacterial reduction was monitored over 24 h (Fig. 2A). Without phage treatment (control), bacterial counts increased from an initial inoculum of 5.0 log₁₀ CFU/mL to 7.3 log₁₀ CFU/mL after 4 h and reached 9.2 log₁₀ CFU/mL after 24 h. In contrast, phage-treated samples showed bacterial counts of 5.2 log₁₀ CFU/mL after 4 h, increasing to 6.1 log₁₀ CFU/mL after 24 h. These correspond to reductions of 2.1 and 3.1 log₁₀ CFU/mL, respectively (*p* < 0.05). However, the treated group’s bacterial populations gradually increased over time, indicating that some regrowth occurred at room temperature. Another experiment assessed phage efficacy under refrigerated storage (4 °C) over 24 h (Fig. 2B). After 4 h, bacterial counts in the untreated control reached 6.4 log₁₀ CFU/mL. In contrast, phage-treated samples were reduced to 3.2 log₁₀ CFU/mL, a 3.2 log₁₀ reduction. After 24 h, the control group reached 6.7 log₁₀ CFU/mL, whereas phage-treated samples decreased further to 3.0 log₁₀ CFU/mL, a 3.7-log₁₀ reduction (*p <* 0.05). It is important to note that no secondary bacterial growth was observed under refrigeration storage for 24 h, demonstrating increased stability and efficiency of phage therapy at 4 °C.

### Antibacterial efficacy of bacteriophage UHP46 in cheese

**Fig. 3:**
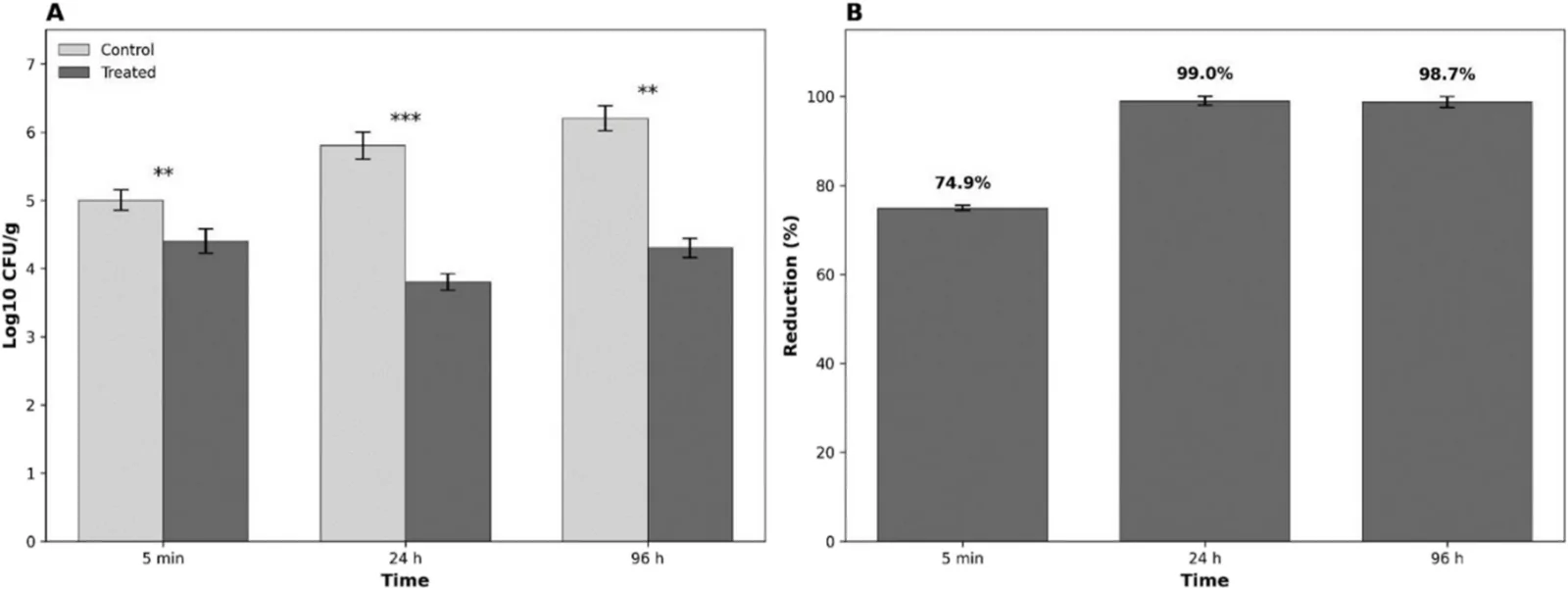
Antibacterial activity of phage UHP46 against *S. aureus* S46 in contaminated cheese during refrigerated storage at 4 °C. (**A**) Viable *S. aureus* S46 counts (log₁₀ CFU/g) measured at 5 min, 24 h, and 96 h post-treatment. (**B**) Percentage reduction compared to the PBS-treated control group

Cheese samples inoculated with *S. aureus* S46 were treated with bacteriophage UHP46 at a final concentration of 1 × 10⁷ PFU/g, resulting in an MOI of 100. After 5 min of treatment, a 74.9% reduction (0.6 log₁₀) in bacterial counts was observed, with levels decreasing from 5.0 log₁₀ CFU/g in PBS-treated controls to 4.4 log₁₀ CFU/g in phage-treated samples (Fig. 3A, B). Following 24 h of storage at 4 °C, *S. aureus* S46 levels in phage-treated samples remained significantly lower than in controls (*p* < 0.001), corresponding to a 99.0% reduction (2.0 log₁₀); control and treated samples measured 5.8 and 3.8 log₁₀ CFU/g, respectively (Fig. 3A). At 96 h, control samples increased to 6.2 log₁₀ CFU/g, while phage-treated samples remained at 4.3 log₁₀ CFU/g, resulting in a sustained reduction of 98.7% (1.9 log₁₀; *p* < 0.01) (Fig. 3A, B). Notably, antibacterial activity increased substantially between the 5 min and 24 h time points, after which reductions stabilized through the 96-h experimental period. For all evaluated time points analyzed, there were lower bacterial numbers in the phage-treated samples compared to the control group, but both showed increasing regrowth tendencies during the 96 h of storage (Fig. 3A, B).

### Antibacterial activity of UHP46 in yogurt under different storage temperatures

**Fig. 4:**
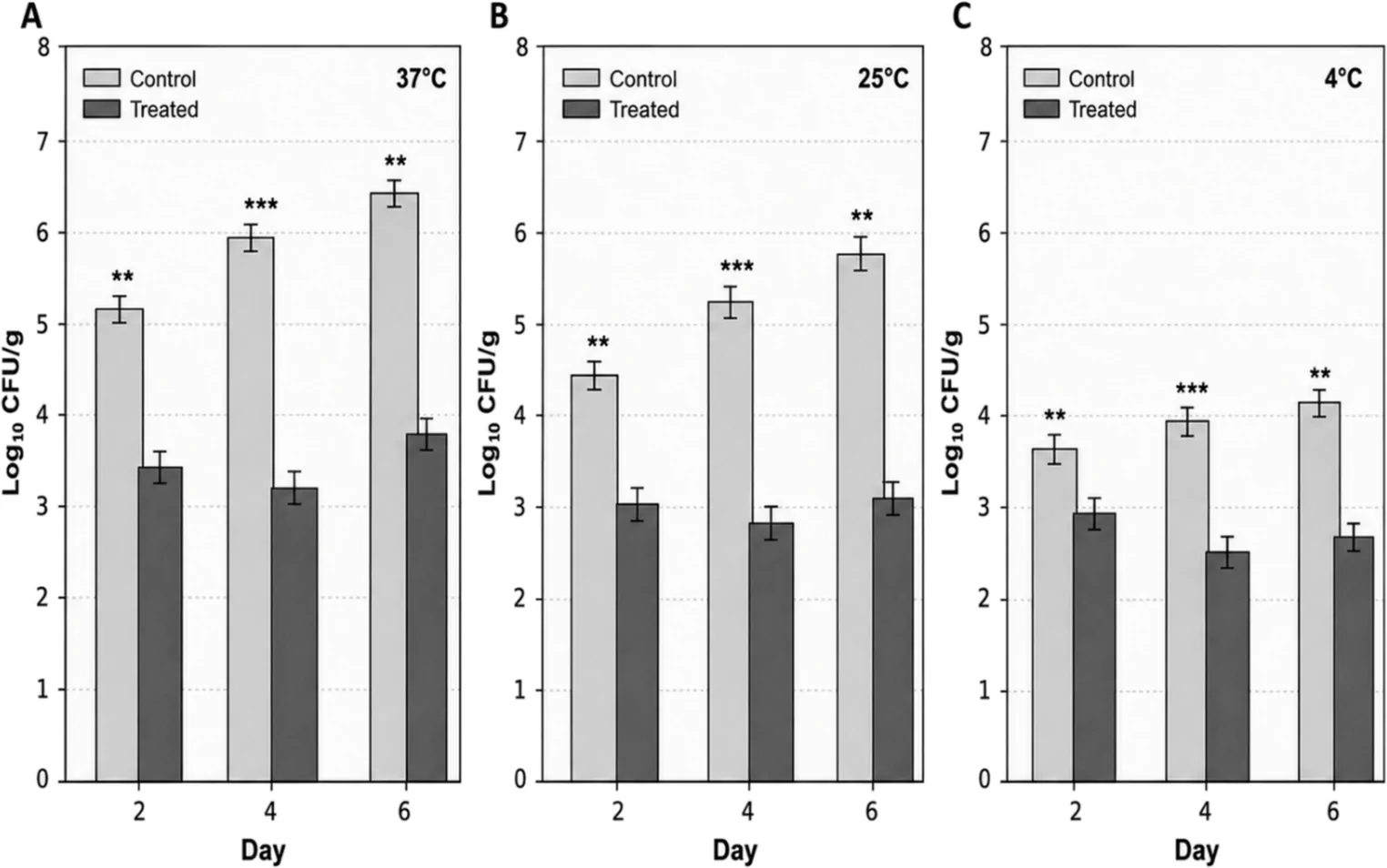
Effect of bacteriophage UHP46 on *S. aureus* S46 in yogurt samples at different incubation temperatures. (**A**–**C**) Bacterial enumeration (log₁₀ CFU/g) in control and phage-treated yogurt at temperatures of (37 °C, 25 °C, and 4 °C), respectively, within a period of six days. Values are expressed as means ± standard deviations (*n* = 3)

The antibacterial activity of UHP46 in yogurt was assessed at three different storage temperatures. Although mean log₁₀ reductions were higher at elevated temperatures, this trend was not statistically significant. At 37 °C, bacterial counts in the untreated control increased progressively from 5.2 ± 0.2 log₁₀ CFU/g on day 2 to 6.1 ± 0.2 and 6.6 ± 0.2 log₁₀ CFU/g on days 4 and 6, respectively (Fig. 4A). Phage-treated samples remained significantly suppressed throughout, with counts of 3.4 ± 0.2, 3.2 ± 0.2, and 3.8 ± 0.2 log₁₀ CFU/g at the corresponding time points (*p* < 0.001). A similar pattern was noticed at 25 °C, although bacteria were less abundant. Control counts increased from 4.5 ± 0.2 log₁₀ CFU/g on day 2 to 5.3 ± 0.2 and 5.8 ± 0.2 log₁₀ CFU/g on days 4 and 6, while phage-treated samples were maintained at significantly lower levels of 3.0 ± 0.2, 2.8 ± 0.2, and 3.1 ± 0.2 log₁₀ CFU/g, respectively (*p* < 0.001; Fig. 4B). Under refrigerated conditions (4 °C) bacterial growth was attenuated in both groups. Control samples increased from 3.6 ± 0.2 log₁₀ CFU/g on day 2 to 4.0 ± 0.2 and 4.3 ± 0.2 log₁₀ CFU/g on days 4 and 6, whereas phage-treated samples remained lower at 2.9 ± 0.2, 2.5 ± 0.2, 2.7 ± 0.2 log₁₀ CFU/g (*p* < 0.01; Fig. 4C). On day 6, UHP46 achieved a log₁₀ reduction of 2.8, 2.7 and 1.6 log₁₀ CFU/g at 37 °C, 25 °C, and 4 °C respectively (Table 2). These results show that UHP46 exhibited high antibacterial activity against *S. aureus* S46 at the various storage temperatures including at refrigerated storage temperature. Log₁₀ reductions and residual viable counts across all three matrices and storage temperatures are summarized in Table 2.

### Effect of storage temperature on phage effectiveness

**Fig. 5:**
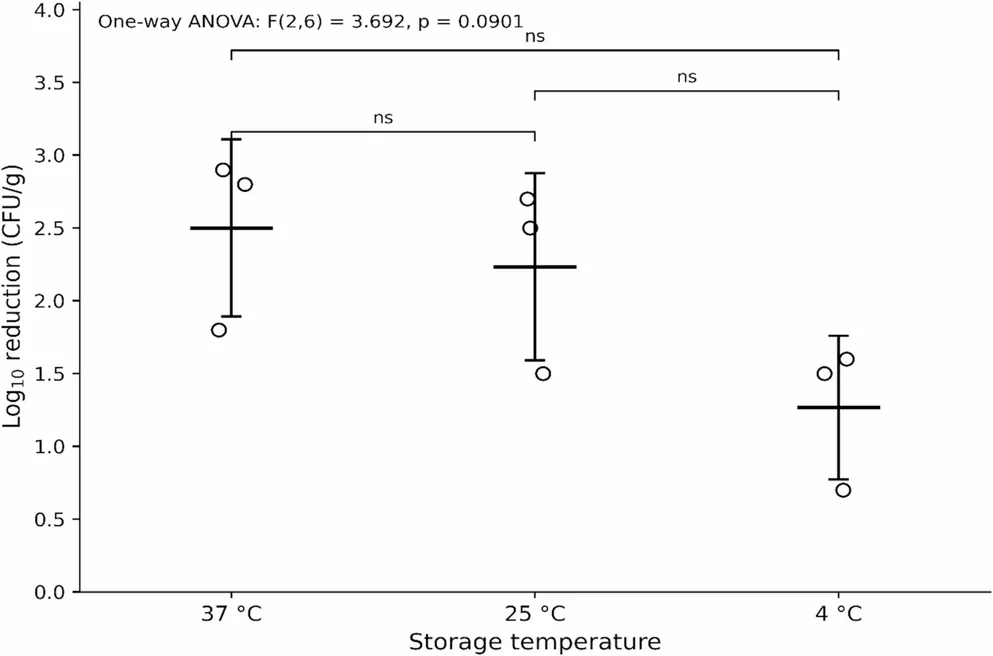
Effect of storage temperature on the antibacterial activity of bacteriophage UHP46 against *S. aureus* S46 in yogurt. Log₁₀ reductions at 37 °C, 25 °C, and 4 °C. Points represent individual observations, and horizontal lines with error bars indicate mean ± SD. Statistical analysis was performed using one-way ANOVA followed by Tukey’s multiple-comparison test. No significant differences were detected among storage temperatures (F(2,6) = 3.692, *p* = 0.0901). ns, not significant

The effect of storage temperature on phage effectiveness was further evaluated (Fig. 5). Log₁₀ reductions in yogurt were compared at 37 °C, 25 °C, and 4 °C using one-way ANOVA followed by Tukey’s multiple-comparison test. The analysis yielded F (2,6) = 3.692, *p* = 0.0901, indicating that the overall effect of storage temperature did not reach statistical significance. Tukey’s post-hoc analysis (Table 1) indicated that there were no statistically significant differences between 37 °C and 25 °C (adjusted *p* = 0.8462), 37 °C and 4 °C (adjusted *p* = 0.0920), or 25 °C and 4 °C (adjusted *p* = 0.1872). Although the mean log₁₀ reduction was numerically greater at 37 °C than at 4 °C, this difference was not significant after adjustment for multiple comparisons.

**Table 1:** Pairwise comparisons of log₁₀ reductions for yogurt stored at different temperatures, analyzed using Tukey’s multiple-comparison test

Comparison	Mean difference	*p*-value	Result
37 °C vs. 25 °C	0.2667	0.8462	ns
37 °C vs. 4 °C	1.2333	0.0920	ns
25 °C vs. 4 °C	0.9667	0.1872	ns

Log_10_ reductions were calculated as the difference between bacterial counts in untreated control and phage-treated samples at each storage temperature and sampling time. Values represent the observed log₁₀ reductions at 37 °C, 25 °C, and 4 °C on days 2, 4, and 6. One-way ANOVA followed by Tukey’s multiple-comparison test was used to compare log₁₀ reductions among storage temperatures. Statistical significance was considered at *p* < 0.05

Comparisons across dairy matrices must account for differences in initial inoculum. Yogurt was inoculated at approximately 2 × 10³ CFU/g, while milk and cheese were inoculated at approximately 1 × 10⁵ CFU/mL and CFU/g, respectively. The direction of the temperature effect varied between matrices, as indicated in Table 2. However, cross-matrix comparisons require caution because the initial inoculum differed between yogurt and milk or cheese. The residual viable count of milk at 4 °C was 3.0 log₁₀ CFU/mL, which was the least and also had the highest net reduction (3.7 log₁₀ CFU/mL) at 24 h. The highest net reductions, however, were found at the higher storage temperatures (2.8 log₁₀ CFU/g at 37 °C and 2.7 log₁₀ CFU/g at 25 °C) for the yogurt, while the lowest number of microorganisms in the treated product occurred at 4 °C (2.7 log₁₀ CFU/g). This apparent paradox comes about because there is net reduction (as measured against an untreated control whose growth also increases with temperature) which represents an even greater suppression of bacterial multiplication, not more extensive inactivation of the initial bacterial population. Cheese, which was evaluated at 4 °C only, showed a kinetic profile different from the other two liquid matrices, with a rapid drop in the viable counts in the first 5 min, reaching its minimum at 24 h, and showing a slight increase at 96 h, which is typical of the kinetics of a matrix with limited phage diffusion. Combined, these data suggest that the residual pathogen load in the treated product is more useful for comparing phage performance in various matrices stored under varying conditions than is net log₁₀ reduction alone.

**Table 2:** Net log₁₀ reduction and residual viable counts of *S. aureus* S46 after UHP46 treatment in milk, cheese, and yogurt at the tested temperature and storage conditions

Matrix	Temp.	Endpoint	Log₁₀ reduction	Treated count
Milk	37 °C	3 h	1.8 log₁₀ CFU/mL	3.8 log₁₀ CFU/mL
Milk	25 °C	4 h/24 h	2.1/3.1	5.2/6.1
Milk	4 °C	4 h/24 h	3.2/3.7	3.2/3.0
Cheese	4 °C	5 min/24 h/96 h	0.6/2.0/1.9	4.4/3.8/4.3
Yogurt	37 °C	Day 6	2.8	3.8
Yogurt	25 °C	Day 6	2.7	3.1
Yogurt	4 °C	Day 6	1.6	2.7

## Discussion

The phage UHP46 was effective in reducing the number of viable cells in milk when the temperature was 37 °C for 3 h by 1.8 log₁₀ CFU/mL, while the phage titer showed a significant increase from 7.2 to 8.8 log₁₀ PFU/mL. The observed rapid replication aligns with the previously determined short latent period of approximately 20 min and a burst size of 27 ± 1 progeny per cell for UHP46, as established by one-step growth curve analysis [26]. These parameters support efficient host lysis and rapid phage propagation within the 3 h incubation period observed in this study. These results are also consistent with the results obtained by García et al. [14], where the lytic action of two phages (phiIPLA35 and phiIPLA88) was capable of inhibiting the growth of *S. aureus* in pasteurized milk, and their efficiency depended directly on temperature and the ratio between host and phage. It was seen that after 4 h and 24 h at 25 °C, the bacterial count in UHP46 reduced by 2.1 and 3.1 log₁₀, respectively, but the bacterial counts in the treated samples were increased with time, indicating some bacterial regrowth. This regrowth has been seen before, and has been explained as the growth of phage insensitive subpopulations within *S. aureus* – phage systems [11].

The susceptibility of the cell survivors was not assessed in the present study, so it is not possible to separate the two possibilities of survival due to resistance or due to the presence of a physiological or spatial refuge and this is a question that still needs to be answered. An increase of approximately 1.4 log₁₀ CFU/mL in untreated milk was observed during the first 4 h at 4 °C. Independent monitoring confirmed that refrigeration temperature remained stable, thereby excluding equipment-related fluctuation. The smaller increase in bacterial counts observed at 4 °C compared to 25 °C likely results from reduced growth activity of *S. aureus* under refrigerated conditions. This initial increase likely reflects residual growth momentum from the actively growing overnight inoculum, as *S. aureus* does not immediately cease division upon transfer to refrigeration. The smaller subsequent increase between 4 and 24 h (6.4 to 6.7 log₁₀ CFU/mL) suggests that bacterial growth had largely plateaued by the later time point. In a refrigerated environment (4 °C), UHP46 exhibited its maximum inhibition capacity, resulting in a 3.7 log₁₀ reduction after 24 h with no secondary growth. The additional reduction observed between 4 h (3.2 log₁₀) and 24 h (3.7 log_10_) at 4 °C was modest compared to the 20-h interval between these measurements. This plateau aligns with the obligate requirement of lytic phages for actively dividing host cells to complete adsorption, replication, and lysis. Under refrigeration, the substantially reduced metabolic activity and division rate of *S. aureus* likely limited the number of susceptible, actively replicating cells available for further phage propagation. This observation aligns with the findings of Fister et al. [12], who reported enhanced phage efficacy under conditions that limit bacterial multiplication. Consequently, further reduction was limited despite the ongoing presence and activity of the phage. This outcome does not indicate a loss of phage efficacy, but rather reflects a phase limited by host replication following the initial rapid reduction phase. The phage KMSP1 reduced *S. aureus* count in pasteurized milk by 8.8 log₁₀ CFU/mL after 24 h, which substantially exceeds the reduction achieved by UHP46 in the current study in which the number of the latter was reduced by 3.7 log₁₀ CFU/mL after 24 h in heat-treated milk at 4 °C. These numbers cannot be compared directly with each other for the two phages. The KMSP1 study utilized a higher MOI than that applied for UHP46 in the present study [21]. While KMSP1 achieved an 8.8-log₁₀ CFU/mL reduction in *S. aureus* in pasteurized milk after 24 h, these outcomes are not directly comparable to the present findings due to differences in inoculum concentration, MOI, phage preparation, and experimental conditions. Consequently, the lower reduction observed with UHP46 should not be regarded as indicative of reduced phage efficacy. A direct comparison would require assessment of both phages under identical experimental conditions. Turning to the solid matrix, UHP46 produced a reduction of 0.6 log₁₀ CFU/g (74.9%) in 5 min for experimentally contaminated hard cheese at 4 °C. After 24 h, this significantly increased to 2.0 log₁₀ (99.0%), and after 96 h, it maintained at 1.9 log₁₀ (98.7%). It is possible that the progressive increase in antibacterial effect from 5 min to 24 h may be attributed to the time needed for the adsorption and reproduction of phages in the cheese, which is more difficult than in aqueous medium [4]. Suppression was maintained through 96 h in both cases, even though growth resumed at that stage, suggesting continuing activity of the phages during refrigeration.

In hard cheese, the present results are similar to those obtained by Bueno et al. [4] with a two-phage cocktail, which resulted in a reduction of 4.64 log₁₀ CFU/g against *S. aureus* Sa9. This larger decrease in that study could be due, at least in part, to the use of a cocktail of phages instead of just one, as a cocktail of phages with different receptor specificities can lead to lower numbers of insensitive survivors [2]. The two studies are also different with regard to host strain, phage type, cheese type, and applied dose, which could have contributed to the difference in outcome, and the contribution of cocktail formulation alone cannot be determined from these data. The first mild decrease at 5 min seen in this study is consistent with findings from phage KMSP1 treated on sliced cheddar cheese, which shows a decrease of 0.6 to 1.4 log₁₀ CFU/g after 30 min [21]. The regrowth that was observed for the phage-treated cheese samples within 96 h is a well-documented behavior that has been noted in solid foods and is believed to be due to the fact that bacteria inside the matrix are inaccessible to the phages [9].

In yogurt, the largest net reductions were observed at 37 °C (2.8 log₁₀ CFU/g) and 25 °C (2.7 log₁₀ CFU/g). However, the lowest residual viable count occurred at 4 °C (2.7 log₁₀ CFU/g), which highlights the distinction between net reduction and absolute endpoint burden. Direct comparison of the reductions observed in yogurt with those in milk and cheese requires caution, as the initial *S. aureus* inoculum in yogurt (approximately 2 × 10³ CFU/g) was lower than that used in milk (approximately 1 × 10⁵ CFU/mL) and cheese (approximately 1 × 10⁵ CFU/g). This variation in starting bacterial load constitutes a limitation when evaluating phage efficacy across different dairy matrices. Mean log₁₀ reductions were higher at 37 °C and 25 °C compared to 4 °C, which aligns with established lytic phage biology indicating that elevated temperature increases adsorption rate, shortens the latent period, and increases burst size [17]. However, this difference was not statistically significant in the present dataset, so the observed trend should be considered suggestive rather than conclusive. These findings align with previously characterized replication parameters of UHP46, which demonstrate efficient replication under permissive host physiological conditions [26]. It has been amply demonstrated that the temperature-sensitive lytic ability follows the same trend; the process is more effective around the optimal growth temperature of the host bacteria because multiplication of the phages relies on the physiological condition of the host cell [11]. Interestingly, even at 4 °C, UHP46 showed significant inhibition over a 6-day duration, where the phage treatment samples were significantly lower than the control samples at each respective time point (*p* < 0.01). Phage activity was maintained in the yogurt matrix under the tested storage conditions. However, the absence of pH measurements for the yogurt used in this study limits direct interpretation of the relationship between yogurt acidity and UHP46 activity [26]. This is a significant finding because the acidic nature of the matrix in fermented dairy products has been identified as one of the challenges that affect phage effectiveness [40]. As a whole, these results suggest that UHP46 retained lytic activity against *S. aureus S*46 on all tested matrices under various storage conditions. The demonstrated effectiveness across milk, yogurt, and cheese matrices highlights a versatility that distinguishes this study from previous investigations, which evaluated phage biocontrol in only a single food type.

Previous research has established the potential of commercial or commercially developed Listeria-specific bacteriophage preparations for food biocontrol. For instance, Leverentz et al. [23] reported that lytic *L. monocytogenes*-specific phages effectively reduced *L. monocytogenes* contamination on fresh-cut fruits.

UHP46 was effective specifically against a single multidrug-resistant *S. aureus* strain (S46) commonly found in bovine mastitis in the dairy farm environment of Pakistan in the present study. This is a limitation of the evidence and not an indication of a broad-spectrum activity since in a previous host range screen UHP46 lysed 4 of the 29 *S. aureus* isolates from mastitis (13.8%) [26]. To confirm clinical relevance for wider application the efficacy of UHP46 should be tested against a broader panel of *S. aureus* strains, including more MDR and MRSA strains. No antibiotic resistance genes, known virulence determinants, nor lysogeny-associated elements were detected in the genome of UHP46, which suggests a strictly lytic phage and a good safety profile for use in food [26]. Its narrow host range also indicates a limited effect on the non-target microflora, which includes the starter cultures used in fermented dairy products, but was not tested here. In addition to the effectiveness demonstrated, several practical issues have to be considered before UHP46 could be used at an industrial level. The production of large-scale phages will depend on the optimization of fermentation, purification and formulation processes to produce stable and uniform phages with consistent titer, with the cost of the production processes being one of the factors that will determine commercial feasibility compared to conventional preservation methods [34]. Regulatory approval is another hurdle, especially in markets like Pakistan, which lack a dedicated regulatory framework for phage-based food biocontrol agents, but this would likely require existing food-safety and biocontrol guidelines to be adapted based on international precedents like the U.S. FDA GRAS approvals for phage products [13, 34]. A further consideration is sensory impact, which is another requirement for any direct intervention in dairy products that would be considered a biocontrol; this was not evaluated in the present study but is an important topic for future evaluation. Last, like any single phage treatment, there is a risk of selecting for subpopulations of phage-resistant *S. aureus* over time, a risk that could be reduced by using phage cocktails or implementing a rotation strategy, instead of single phages [2]. Future research should include evaluation of the efficacy of UHP46 against additional *S. aureus* strains, testing its efficiency in conjunction with other biological control compounds, and assessing its ability to inhibit staphylococcal toxin production in infected dairy products.

## Conclusion

The bacteriophage UHP46 exhibited strong inhibition of *S. aureus* S46 in the tested dairy matrices, including milk, cheese, and yogurt, across various storage temperatures. This naturally derived biocontrol agent demonstrates potential for further evaluation in dairy applications without reliance on antibiotics. As previously reported, genomic characterization of UHP46 showed that no antibiotic resistance genes, previously known virulence factors or DNA regions associated with lysogeny were present, indicating a safety profile consistent with a strictly lytic phage biology. The present study assessed the antibacterial efficacy rather than safety, but more regulatory-grade characterization would be needed before it could be used commercially. Further studies are required of the efficacy of UHP46 as a phage cocktail, and to evaluate the efficacy against different *S. aureus strains* and different MRSA isolates, as well as under commercial processing conditions.
